# The three-legged stool of evidence-based practice in eating disorder treatment: research, clinical, and patient perspectives

**DOI:** 10.1186/s12916-016-0615-5

**Published:** 2016-04-14

**Authors:** Carol B. Peterson, Carolyn Black Becker, Janet Treasure, Roz Shafran, Rachel Bryant-Waugh

**Affiliations:** Department of Psychiatry, University of Minnesota Medical School, F282/2A West, 2450 Riverside Avenue South, Minneapolis, MN 55454 USA; The Emily Program, St. Paul, MN USA; Department of Psychology, Trinity University, San Antonio, TX USA; Department of Psychological Medicine, IOPPN, King’s College, London, UK; Institute of Child Health, University College, London, UK; Great Ormond Street Hospital for Children, London, UK

**Keywords:** Anorexia nervosa, Binge eating disorder, Bulimia nervosa, Eating disorders, Treatment outcome

## Abstract

**Background:**

Evidence-based practice in eating disorders incorporates three essential components: research evidence, clinical expertise, and patient values, preferences, and characteristics. Conceptualized as a ‘three-legged stool’ by Sackett et al. in 1996 (BMJ), all of these components of evidence-based practice are considered essential for providing optimal care in the treatment of eating disorders. However, the extent to which these individual aspects of evidence-based practice are valued among clinicians and researchers is variable, with each of these stool ‘legs’ being neglected at times. As a result, empirical support and patient preferences for treatment are not consistently considered in the selection and implementation of eating disorder treatment. In addition, clinicians may not have access to training to provide treatments supported by research and preferred by patients. Despite these challenges, integrating these three components of evidence-based practice is critical for the effective treatment of eating disorders.

**Discussion:**

Current research supports the use of several types of psychotherapies, including cognitive-behavioral, interpersonal, and family-based therapies, as well as certain types of medications for the treatment of eating disorders. However, limitations in current research, including sample heterogeneity, inconsistent efficacy, a paucity of data, the need for tailored approaches, and the use of staging models highlight the need for clinical expertise. Although preliminary data also support the importance of patient preferences, values, and perspectives for optimizing treatment, enhancing treatment outcome, and minimizing attrition among patients with eating disorders, the extent to which patient preference is consistently predictive of outcome is less clear and requires further investigation.

**Summary:**

All three components of evidence-based practice are integral for the optimal treatment of eating disorders. Integrating clinical expertise and patient perspective may also facilitate the dissemination of empirically-supported and emerging treatments as well as prevention programs. Further research is imperative to identify ways in which this three-legged approach to eating disorder treatment could be most effectively implemented.

## Background

Increasingly, clinicians in all areas of health services are being encouraged to engage in evidence-based practice. Evidence-based practice encourages clinicians to (1) use the best available research evidence in conjunction with (2) clinical expertise, whilst (3) considering patients’ characteristics, values, and circumstances to inform care [1]. These three aspects have been referred to as the ‘three-legged stool’ of evidence-based practice [1, 2].

Many clinicians and researchers, however, appear to consider the construct ‘evidence-based practice’ as synonymous with ‘evidence-based treatments’ or ‘empirically-supported therapies’. These latter constructs are solely concerned with research evidence supporting particular interventions, whereas evidence-based practice has the additional strands of clinical expertise and patient characteristics. Nevertheless, clinicians often appear to discount research evidence – the first leg of the stool—for a variety of reasons, including perceived differences between samples in clinical trials and those in routine clinical settings [3]. Within the area of eating disorders in particular, this disregard has led to the majority of patients not receiving treatment demonstrated to be efficacious in randomized controlled trials [4]. Indeed, figures from the UK suggest that only 4 % of family physicians used published clinical guidelines in the treatment of eating disorders [5].

The significant reservations about data from clinical research studies, coupled with concern about specific techniques such as those used in cognitive-behavioral therapy (CBT) [6], suggest that decision-making regarding treatment selection is often guided by the remaining ‘legs’ of the three-legged stool. For example, surveys indicate that some therapists tend to rely on their clinical experience or that of their peers in their clinical decision-making rather than on research [7]. However, clinical experience has been shown to be subject to a number of significant biases [8] and basing decision-making purely on clinical experience is likely to contribute to ‘therapist drift’ from protocols that may negatively impact therapy and make it difficult to maintain treatment integrity [9]. On the other hand, limitations in research, including the lack of data on clinical interventions for some conditions, can complicate the extent to which empirical findings can inform treatment. With regards to the final leg of the stool, namely the consideration of patient characteristics, values, and circumstances to inform care, relatively little research has been conducted on the role of patient preferences in clinical decision-making in the treatment of eating disorders, but the emphasis on patient choice indicates that clinicians are increasingly aware of the importance of patient preferences and values in determining treatment strategies.

The issues arising from using only one of the ‘legs’—i.e. research evidence or clinical judgement or patient values—to guide clinical decision-making are multiple and profound. Further, understanding that the foundations of evidence-based practice are grounded in the combination of each of these three elements is the necessary first step for the improvement of patient treatment as well as towards the integration of all three ‘legs’ of the stool in evidence-based practice. Regular progress monitoring and feedback using tools developed from, and used by, research can help guide clinical decision-making and may also influence patient values [10, 11]. The implementation of flexible treatment manuals to facilitate fidelity to the protocol allows clinicians to be fully creative and to personalize treatment to clients, thus harnessing clinical judgement and skills [12]. In order to achieve this, changing the methods by which research findings are communicated to increase their relevance to clinicians is fundamental. In addition, ensuring that participants studied in research are representative of clinical samples in terms of comorbidities, ethnicity, socioeconomic status, and other variables, as well as the publishing of negative results, would help bridge the gap between science and practice.

The inherent problems in the use of evidence-based practice are not specific to eating disorders or mental health; rather, they impact across multiple spheres, including medicine and public health [13]. Nevertheless, in eating disorders in particular, there is increasing interest in attempting to understand and resolve some of the difficulties with a view to improving clinical outcomes. Such growing interest led to a symposium being convened at the London 2015 Eating Disorders Conference in March 2015, entitled The Three-Legged Stool of Evidence-Based Practice: Research, Clinical, and Patient Perspectives, with the aim to implement each of the ‘legs’ of the stool in evidence-based practice. The three ‘legs’ are summarized below.

## Discussion

### Research evidence

As specified by Sackett et al. [1], the research ‘leg’ of the evidence-based stool relies on the most current empirical evidence of treatment efficacy and effectiveness based on randomized controlled trials (ideally, double-blinded), meta-analyses, systematic reviews, case series, and other types of research designs. Determining treatment based on findings from research investigations, particularly using well-designed studies, minimizes the occurrence of clinical biases in treatment selection [3, 8] and helps ensure that patients receive the optimal treatment based on current findings.

Current research data suggest that cognitive behavioral approaches, including enhanced CBT (CBT-E) [14] as well as CBT-guided self-help, have been most efficacious among adults with bulimia nervosa and binge eating disorder; data supporting the use of CBT for adults with anorexia nervosa and adolescents with eating disorders are more inconsistent [15–22]. Similarly, the use interpersonal therapy (IPT) to treat eating disorder symptoms is primarily supported in adults with bulimia nervosa and binge eating disorder, with preliminary treatment outcome data suggesting that dialectical behavior therapy (DBT) may also be promising in these populations [15–18]. For adolescents with anorexia nervosa or bulimia nervosa, family-based treatments (FBT) have been found to be associated with significant improvements, although comparable adolescent-focused treatments (AFT) have also shown promise [15, 16, 20, 21, 23]. Finally, selective serotonin reuptake inhibitors and tricyclic antidepressants have been associated with reductions in eating disorder symptoms in bulimia nervosa and binge eating disorder with more modest support for the use of topiramate; in contrast, minimal empirical evidence has been found for the use of medications in the treatment of anorexia nervosa [16, 20]. In randomized trials, the added benefit of pharmacotherapy when combined with psychotherapy has received inconsistent support for the treatment of eating disorders, with a few studies finding a significant effect for combined treatment [24] and others finding minimal additive effects [25, 26].

Although potentially useful in guiding treatment selection, consistent predictors of treatment outcome have not been identified in eating disorders. One notable exception is rapid response in CBT: among individuals with bulimia nervosa and binge eating disorder, significant symptomatic reduction in the first month has been associated with better outcome at end of treatment [27, 28]. This robust finding has important clinical implications, especially in the context of stepped care models. More recently, increasing attention has been focused on identifying treatment-specific moderators and mediators that can be used to optimize treatment selection [29]. Preliminary support for several treatment-specific moderators has been found in randomized controlled eating disorder treatment studies. More specifically, adults with severe and enduring anorexia nervosa who were older, classified as binge eating and purging subtype, and with higher levels of depression and global eating disorder psychopathology showed greater improvement in CBT than in specialist supportive clinical management [30]. For adolescents with anorexia nervosa who received FBT or AFT, those with higher baseline levels of eating-related psychopathology and obsessionality benefited more from FBT than AFT at end of treatment [31]. Adolescents with bulimia nervosa who reported higher levels of purging at baseline, as well as those who were younger, showed greater improvement in FBT than in supportive psychotherapy [32]; a previous study also observed that FBT was associated with better outcomes for participants who reported lower scores on global eating disorder psychopathology (e.g. eating, weight, and shape concerns) [33]. Additionally, a recent study found that participants with symptoms of bulimia nervosa who reported higher levels of stimulus seeking and affective instability showed more improvement in integrative cognitive-affective therapy than in CBT; individuals lower in stimulus seeking had better treatment outcome in CBT than in integrative cognitive-affective therapy [34]. Another recent study observed that, for participants with both bulimia nervosa and borderline personality disorder, the broad version of CBT-E was associated with better outcome compared to the focused version for those who reported high levels of baseline emotional and interpersonal distress; participants with lower levels of emotional and interpersonal distress reported more improvement with the focused version of CBT [35]. For binge eating disorder, a randomized trial found that those with avoidant personality disorder and early onset overweight and dieting had poorer outcome in an active control condition than in DBT [36]. Overvaluation of shape and weight has also been identified as a treatment moderator in binge eating disorder as it was associated with better outcome in CBT compared to psychopharmacological treatment, even when controlling for negative affect [37]. In the same study, younger patients had better outcome with fluoxetine, whereas higher levels of baseline negative affect and binge eating frequency and lower levels of self-esteem were associated with better outcome in CBT; in addition, older age of onset of binge eating disorder was associated with more rapid improvements in CBT [37]. Another randomized trial for the treatment of binge eating disorder found that higher levels of eating disorder psychopathology and lower self-esteem were associated with better outcome in CBT and IPT compared to CBT-guided self-help [38]. Finally, in a heterogeneous eating disorder sample, participants with lower self-esteem and higher levels of mood intolerance, interpersonal difficulties, and clinical perfectionism showed improved response to the broad version of CBT-E compared to the focused version [39].

In contrast to the growing list of potential moderators of treatment, identification of mediators remains more elusive. Although potential treatment mediators, including motivation [40] and obsessive compulsive symptoms [41], have been observed in naturalistic studies, significant mediators have not been identified in randomized treatment trials [e.g. 31]. In summary, rapid response in CBT appears to be a robust predictor of outcome; alternative or adjunctive treatments should be considered for patients with eating disorders who do not show initial improvement with CBT. Although preliminary data support several treatment-specific moderators, replication and robust data are needed to implement these empirical findings in clinical settings for treatment selection. Further research is needed to identify treatment mediators.

Empirical examinations of emerging treatments for eating disorders are underway, including couples and family treatment for adult patients, carer interventions, cognitive remediation, mindfulness approaches, emotion-focused therapies, acceptance and commitment therapy, and additional pharmacological agents. In addition, innovative treatment delivery is being investigated using the internet and mobile phones. Empirically-informed stepped-care and staging models may also improve treatment outcome. An increasing focus on identifying causal and maintenance mechanisms and an understanding of their psychological and neurobiological manifestations will ideally facilitate treatment targets among emerging treatments as well as strengthen the likelihood of identifying moderators that can be used to determine which treatments work optimally for which patients [29, 42]. Another critical consideration is the dissemination and implementation of evidence-based treatments given that clinicians often site the lack of training and expertise for their decreased likelihood to use these interventions [43]. Thus, scalability is an important feature of evidence-based treatments.

Although the importance of using research to guide treatment cannot be overemphasized, limitations in treatment research highlight the value of considering empirical findings in the context of both clinical expertise and patient preferences and values. First, despite impressive effect sizes in many trials, eating disorder treatment outcome studies are generally characterized by significant rates of attrition, relapse, and non-remission [16, 18, 19]. In addition, with the exception of CBT-E [14], most eating disorder treatments have been developed for one type of eating disorder (e.g. bulimia nervosa, anorexia nervosa, or binge eating disorder) and have not been tested in samples that include subthreshold or mixed diagnostic symptoms despite the high prevalence of these cases in clinical settings [44, 45]. Many trials have not included males, youth, or individuals with certain types of comorbid psychopathology (e.g. substance use disorder), or ethnically diverse samples. Notably, although best practices often utilize inpatient, residential, partial, day treatment, and intensive settings, minimal data are available for treatment delivered in settings other than outpatient settings. Additionally, the rigors of the scientific process make the incorporation of novel and experimental treatments into the evidence base slow. Therefore, the use of clinical expertise and patient values and preferences along with empirical support in determining eating disorder treatment has a number of advantages. First, the combined perspective will potentially increase ecological validity and identify treatments that may not be acceptable to patients, regardless of empirical support. Further, the additional legs will contribute to the development of novel treatments that can eventually be tested and added to the body of evidence-based treatments. Finally, the use of clinical expertise along with patient preferences and values can potentially help guide treatment dissemination and prevention strategies.

### Clinical expertise

Clinical expertise remains a critical component of eating disorder treatment given that several questions and concerns remain inadequately addressed by the existing research literature. For instance, even the diagnosis or the ‘what’ of an eating disorder is of limited utility for guiding management as additional aspects such as how much, why, and with what also play crucial roles. In this context, clinical expertise and assessment skills are essential. Diverse psychological comorbidities are also embedded within the eating disorder spectrum, and physical state ranges from severe undernutrition to hyperalimentation. Moreover, the case formulation needs to include age, stage, and the psychosocial context [46]. Thus, matching diagnosis to evidence-based treatment while considering patient characteristics and preferences is a complex art.

This complexity is recognized, in part, within the DSM-5 [47] description of anorexia nervosa, which now uses a body mass index scale to provide a range of severity. However, although body mass index is a proxy measure of risk, other factors should also be considered, such as the rate of weight loss, fluid and electrolyte disturbance, markers of metabolic and cardiovascular risk, and pubertal stage, sex, ethnic group, duration of illness, and comorbidity. There are wide cultural variations in how cases with high risk are matched to service/treatment as well as the form and content of such treatment. For example, cultural factors may impact the extent to which treatment is legally mandated as well as whether patients are able to refuse treatment and the selection of treatment settings (e.g. outpatient versus hospital based). The field has moved from the position advocated by Gull 150 years ago, where everyone was admitted to hospital because “*family and friends are the worst attendants*” [48], to involving the family and limiting inpatient care to those at extreme risk. In addition, the goal of admission for adolescent cases is to ameliorate risk rather than attain full weight restoration.

Time is of the essence. Prepubertal cases of anorexia nervosa have more acute and complex longer term risks. In addition, the duration of untreated illness (either the result of delay in presentation or because of a failure to respond to treatment) is a key prognostic indicator [49]. One hypothesis is that neuroadaptive changes to persistent starvation and abnormal eating behaviors result in treatment resistance [46, 50]. This conceptualization has fostered the development of brain-directed treatments that use learning to remediate inflexibility and detailed thinking or train attention away from specific eating disorder salient or more general threat cues as well as neuromodulation to modify mood or habitual behaviors.

The diversity in causal and maintaining factors is now accepted, although how this complexity of factors should be assessed and addressed is still in the early stages. Many evidence-based treatments are modularizing treatment so that specific targets can be addressed. For example, CBT-E has three optional treatment modules that address interpersonal difficulties, core low self-esteem, and clinical perfectionism [14]. The Maudsley Model of Anorexia Nervosa Treatment for Adults (MANTRA) also includes a case formulation and nine treatment modules, including interpersonal relationships, emotional regulation, and cognitive remediation [51]. Despite these clinical and scientific advances in modular treatments, however, dissemination and implementation of evidence-based treatments are essential to enable clinicians to utilize these state-of-the-art interventions in selecting and personalizing clinical interventions. In addition, the extent to which clinicians are trained to provide treatments that are selected and preferred by patients is a critical consideration in the context of implementing evidence-based practice.

In conclusion, we are still at the early stages of personalized care in eating disorders; however, with advances in defining brain-specific pathways from genetics and neuroscience, clinical expertise is essential in terms of tailoring treatment.

### Patient values and preferences

The third leg of the evidence-based stool consists of patient values and perspectives [1]. With regards to mental health, researchers typically study patient values and perspectives under the headings of patient preferences and expectancies; this section explores three questions with respect to these factors. First, do patient preferences and expectations actually matter in eating disorder treatment? Second, can providers improve outcome expectancies (and, likely, preferences)? Third, what aspects of eating disorder psychopathology complicate the reliance on patient preferences (e.g. desire to remain underweight)?

Patient preferences are best understood as something desired by patients [52]. For instance, patients might prefer psychotherapy over medication, a particular type of psychotherapy (e.g. behavioral), or a particular type of therapist (e.g. based on sex, age, or ethnicity). Patient expectancies involve predictions. Role expectancies consist of what patients anticipate will happen in treatment (e.g. the process); outcome expectancies include predictions about the effectiveness of treatment and the probability of improvement [53, 54]. Outcome expectancies are a form of response expectancies, which underpin both the placebo and nocebo responses, and play a key role in the efficacy of many treatments [55].

With regards to the first question about the importance of patient preference, we contend that patient preferences and expectations likely influence eating disorder treatment for two reasons. First, eating disorders are associated with high rates of attrition [56, 57], and research suggests that failure to attend to patient preferences in psychotherapy increases dropout [57, 58]. Using non-eating disorder psychiatric samples, several studies have observed that matching treatment with patient preferences reduced attrition for a range of disorders (e.g. anxiety, depression, substance abuse) and may be associated with improved outcomes [59–61]. Further, some evidence suggests that lower outcome expectancies also increase attrition [62]. However, other randomized controlled trials of psychiatric samples have found no significant effect for treatment preferences on attrition or outcome [63], with others suggesting that the impact may depend on the strength of the preference [64]. The extent to which findings from other types of psychiatric disorders can be generalized to eating disorders is unclear, particularly given that eating disorders are often associated with high rates of treatment refusal [65].

Despite the lack of clarity regarding the consistent impact of patient preferences on treatment outcome in psychiatry research, an additional consideration is the extent to which data support an association between outcome expectancies and treatment outcome [54]. In other words, patients who expect to do well with a given treatment are more likely to benefit than those who hold lower expectations. Importantly, outcome expectancies have long been viewed as a key common factor for good psychotherapy outcome [62, 66]. Constantino et al. [67] found that outcome expectancies significantly predicted treatment alliance in eating disorders, which is important as therapeutic alliance itself has been consistently found to predict treatment outcome [68–70]. Although the relationships between expectancies, patient preferences, and treatment outcomes are unclear, some evidence suggests that preferences for CBT versus pharmacotherapy moderate response to both interventions [61, 71]. However, further research is needed. To summarize, patient preferences and expectancies are likely to have an impact in eating disorder treatment since both appear to influence attrition; outcome expectancies also consistently appear to influence treatment outcome and some preferences appear to moderate outcome. Nonetheless, research addressing these topics in eating disorder samples is needed.

With regards to the second question about the extent to which clinicians can influence outcome expectancies, minimal research has been conducted with eating disorder patients. Yet, as noted above, outcome expectancies have been discussed as a key component to psychotherapy; indeed, some writers view psychotherapy as “*inextricably linked with the manipulation and revision of patients’ expectations*” ([72], p. 671). For instance, CBT provides a rationale for treatment, which theoretically improves credibility and provides a reason to believe that treatment can work. CBT therapists also typically provide patients with an overview of research support for CBT, which leads to further evidence of treatment effectiveness. Similarly, research clearly shows that outcome expectancies influence the efficacy of pharmacotherapy and such studies require manipulation of expectancies [73]. Thus, we contend that there appears to be evidence in both the psychotherapy and pharmacotherapy fields that suggests outcome expectancies for treatment can be improved; further, improved outcome expectancies may drive some shift in preferences. Kirsch [74] suggests the following strategies for enhancing outcome expectancies: (1) create a strong therapeutic relationship, which improves confidence in the provider and what they say, (2) foster confidence in treatment effectiveness, anticipation of substantial change, and expectation that change will occur slowly so that faster than expected change can positively snowball expectancies, along with an attitude that one must work to achieve change.

A third consideration is the complexity of relying on patient preference given the nature of eating disorder psychopathology and the frequency of treatment refusal and ambivalence among individuals with eating disorders, particularly anorexia nervosa [65]. Indeed, the fear of weight gain and overvaluation of shape and weight that characterize eating disorders [47] may result in the rejection of interventions that serve as the basis of evidence-based treatment (e.g. self-monitoring, meal planning, behavioral exposure, consumption of feared foods, weight restoration to improve medical and cognitive status, involvement of family members). Alternatively, eating disorder psychopathology may influence the preference of treatments that potentially lead to weight loss (e.g. topiramate as a medication for binge eating). Given that these clinical features may, in fact, contribute to the maintenance of eating disorders, incorporating patient preference into treatment selection among individuals with eating disorders may be especially problematic and complicate treatment outcome. An additional consideration is that patients may express preferences for treatments that have been found to be ineffective in research trials. This scenario is especially problematic because it enables the patient to believe that they are engaged in treatment and yet they are not actually receiving an effective intervention. In evidence-based practice, clinicians must navigate the complexity of respecting patient desires while also relying on data and expertise to collaboratively identify more potentially efficacious treatment options. Finally, recognizing the potential influence of therapist factors on treatment selection, including anxiety [75], and which may interact with expressed patient preference, can help guide the clinician in treatment selection by recognizing potential vulnerabilities and biases.

In summary, minimal research has been conducted on the effects of treatment preference on outcome and attrition among patients with eating disorders; nevertheless, data from non-eating disorder psychiatric samples provide some support for the potential positive impact of patient preference on treatment outcome. The frequent occurrence of treatment refusal and ambivalence among individuals with eating disorders makes future investigations to determine the impact of treatment preference in eating disorders especially important.

## Summary

In summary, each ‘leg’ of evidence-based practice provides an integral component of eating disorder treatment. The three-legged stool nonetheless requires extensive data, expertise, and knowledge to ensure that each leg is equally sturdy. Research indicates that, for the treatment of bulimia nervosa and binge eating disorder, CBT and IPT have strong empirical support whereas DBT and certain types of medications have modest support. FBT has been found to be efficacious in children and adolescents with anorexia nervosa and bulimia nervosa, for whom timely interventions are particularly crucial. The identification of reliable treatment predictors, moderators, and mediators will be especially useful in determining optimal treatment. Clinical expertise is essential for tailoring interventions for both existing and emerging treatments as well as determining care when empirical data are lacking. Preliminary data support the potential importance of patient perspectives, values, and preferences in optimizing treatment outcome, although the clinical features of eating disorders may complicate this process among individuals who are frightened of or ambivalent about treatment. Empirical examinations of emerging treatments may increase the breadth of treatment options supported by data, although the use of clinical expertise and patient preferences and values may help to develop novel treatments, determine treatment when evidence-based interventions have not been identified, and facilitate dissemination and prevention efforts. Finally, future investigations are critical, particularly to determine more efficacious eating disorder treatments, to examine patient preferences in the context of randomized clinical trials, and to identify effective treatment dissemination and implementation strategies to better serve clinicians in the community providing care to patients.
